# Simple Fabrication of Gold Nanobelts and Patterns

**DOI:** 10.1371/journal.pone.0030469

**Published:** 2012-01-23

**Authors:** Renyun Zhang, Magnus Hummelgård, Håkan Olin

**Affiliations:** Department of Natural Sciences, Engineering and Mathematics, Mid Sweden University, Sundsvall, Sweden; Massey University, New Zealand

## Abstract

Gold nanobelts are of interest in several areas; however, there are only few methods available to produce these belts. We report here on a simple evaporation induced self-assembly (EISA) method to produce porous gold nanobelts with dimensions that scale across nanometer (thickness ∼80 nm) and micrometer (width ∼20 µm), to decimeter (length ∼0.15 m). The gold nanobelts are well packed on the beaker wall and can be easily made to float on the surface of the solution for depositing onto other substrates. Microscopy showed that gold nanobelts had a different structure on the two sides of the belt; the density of gold nanowires on one side was greater than on the other side. Electrical measurements showed that these nanobelts were sensitive to compressive or tensile forces, indicating a potential use as a strain sensor. The patterned nanobelts were further used as a template to grow ZnO nanowires for potential use in applications such as piezo-electronics.

## Introduction

Gold nanoparticles [1], nanowires/nanorods [2], nanofilms [3], representing 0D, 1D and 2D gold nanostructures, have been intensively focused in the past decade. Chemical and physical methods have been developed to synthesize these nanostructured materials toward different applications, including electronics, sensors, and nanomedicines, etc. A less well-investigated structure is the nanobelts, which is an intermediate morphology structure with dimension between 1D and 2D and is defined as 1.5D [4]. Structurally, nanobelts are important because they may be the ideal system to investigate the dimensionally confined transport behavior [5]. From an application point, nanobelts are excellent to make functional devices.

Recently, sonochemical and other routes have been developed to synthesize high purity gold nanobelts using glucose [6], surfactant [7], or polyvinylpyrrolidone (PVP) and n-pentanol [8]. Besides, gold belts can also be created in a Fe matrix, based on a directional eutectoid transformation followed by selective phase electrochemical dissolution of Fe [9]. Porous gold nanobelts can also be synthesized by transformation from metal-surfactant complex precursor nanobelts [10]. All these methods can be used to synthesize single crystallized gold nanobelts with L (length)×W (width)×T (thickness) of (∼20 µm)×(∼250 nm)×(∼40 nm).

Although these methods can efficiently produce gold nanobelts by simple procedures, there are some problems, such as the presence of extra reagents and the limitation of the belt length [6], [7]. The presence of extra reagents requires extra rinsing to clean the nanobelts, and the limitation in length restricts the applications area, for example to produce a two terminal electronic device requires long enough nanobelt to get separated electrodes.

In this paper, we report on a simple method, free from extra reagents, to create porous gold nanobelts with L×W×T of (∼0.15 m)×(∼25 µm)×(∼80 nm), based on the evaporation induced self-assembly (EISA) of gold nanoparticles [11], [12]. These monolayered gold nanobelts are grown on the beaker wall during the deposition process, and can easily be made to float on the surface of the solution by tapping the beaker wall, making it easy to deposit them on any kind of substrates. The belt structure is grown due to the temperature difference between the solution and the surrounding air, forming well packed patterns. These gold nanobelts were sensitive to compressive and tensile forces measured as changes in the source/drain (IV) current, suggesting a potential application as strain sensors. The patterned gold nanobelts were further used to grown patterned ZnO nanowires. Moreover, these well packed gold nanobelts can be used to make gold nanoparticle patterns by annealing the gold belts patterns at 1100°C to melt the gold nanowires and form gold nanoparticles.

## Materials and Methods

### Synthesis of gold nanoparticles

Gold nanoparticles were synthesized by the reduction of HAuCl_4_ (Sigma, analytical grade) with citric acid (Sigma, analytical grade). Briefly, heat 100 ml 0.01 wt % HAuCl_4_ to boiling point, then add 3 ml citric acid and keep boiling for 5 min. The nanoparticles were with an average size of 10 nm. A TEM image of the synthesized gold nanoparticles is shown in Figure S1.

### Growth of gold nanobelts and gold nanoparticle patterns

Gold nanobelts were deposited on the beaker wall using an EISA method similar to the deposition of gold nanofilms [11], [12]. However, the difference is in the pre-treatment of synthesized gold nanoparticles. The synthesized gold nanoparticles were first heated to 40°C, 50°C or 70°C and kept for 1 h and then taken out and kept at ambient temperature for 4 h for deposition with a humidity of 23%. To float the gold nanobelts on the beaker wall, distilled water was added over the deposition line. To transfer the gold nanobelts to a silicon wafer or rubber sheet, a cleaned silicon wafer was put under the gold film, and then deposited by retracting the wafer or rubber from the solution with a retracting speed of 0.5 cm/s.

Gold nanoparticles patterns were fabricated by annealing the gold nanobelts on silicon wafer at 1100°C for 10 min in a muffle furnace.

### Growth of ZnO nanowires

The ZnO nanowires were grown as described by Eustis et al [13]. Briefly, 1∶1 mixed (by mass) ZnO powder and graphite was placed at one terminal of small quartz tube centered in a tube furnace. A small piece of Si wafer coated with gold nanobelts was placed 1 cm from the mixture. To grow ZnO nanowires, the tube furnace was heated to 913°C at the speed of 100°C per min and hold at this temperature for 15 min. The pressure inside the tube was controlled at 9 mBar.

### Characterizations

Atomic force microscope (AFM) imaging was performed on an EasyScan 2 microscope (Nanosurf) using contact mode. Scanning electron microscopy (SEM) imaging was done using an EVO50 microscope (Zeiss) with SE1 Mode. Transmission electron microscope (TEM) characterization was performed on a JEOL 2000FX (JEOL) microscope. Electric properties were carried out on a micromanipulator 1800 wafer probe station (Micromanipulator), using two stainless wires as source and drain electrodes. For measuring single gold nanobelts, the distance between the two electrodes was 2 mm, while the electrodes were contacted to silver glue with 1 cm distance for strain sensing measurements on a rubber.

## Results and Discussion

The gold nanobelts in our experiments were self-assembled from gold nanoparticles that were synthesized by the reduction of HAuCl_4_ with citric acid. The growth process is similar to the deposition of gold nanofilms on the beaker wall [11], [12]. However, the difference is the pre-treatment of the gold nanoparticles solution, which was pre-treated at 40°C, 50°C and 70°C for 1 h in an oven. After the pre-treatment, the solution was taken out and kept at ambient temperature for 4 h to grow the nanobelts. Gold nanobelts were observed on the beaker wall caused by the self-assembly of gold nanoparticles [11], [12]. To float the gold nanobelts on the solution surface, doubly distilled water was added over the initial deposition line. After that, the gold nanobelts could be easily transferred to a silicon/silicon dioxide wafer or a rubber wafer by placing the substrate under the gold nanobelts and then retracting the substrate from the solution.

**Figure 1:**
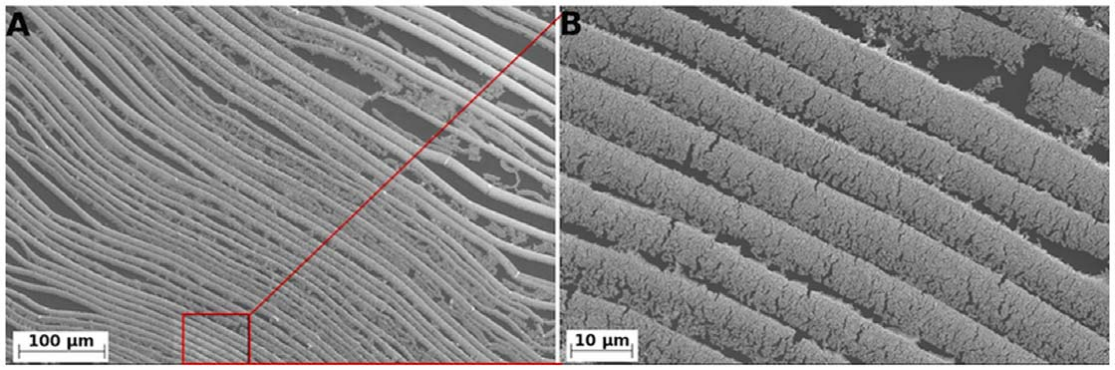
SEM of gold nanobelts, showing small (A) and large (B) magnifications.

SEM results showed that the pattern grown by pre-treating at 40°C only were not obviously different from the ones grown at room temperature, which might due to the small temperature difference between the solution and the surrounding air. When the temperature solution was above 50°C, fully developed gold nanobelts were observed. (A SEM image of gold nanobelts grown at pre-treatment of 40°C and 70°C were shown in Figure S2 and S3). Figure 1 shows SEM images of the deposited gold nanobelts on a silicon wafer, indicating a patterned gold nanobelt film with an average width of 12 µm. Although the nanobelts were closely packed, they could be dispersed by tapping the beaker wall when the nanobelts were floating on the surface of the solution. From the SEM image, it is clear that there cracks appeared in these gold nanobelts, which might have been created during the retracting process where the fluid might generate tensile force breaking some parts of the gold nanowire networks. However, most of the nanobelts were intact, according to IV measurement as described below.

**Figure 2:**
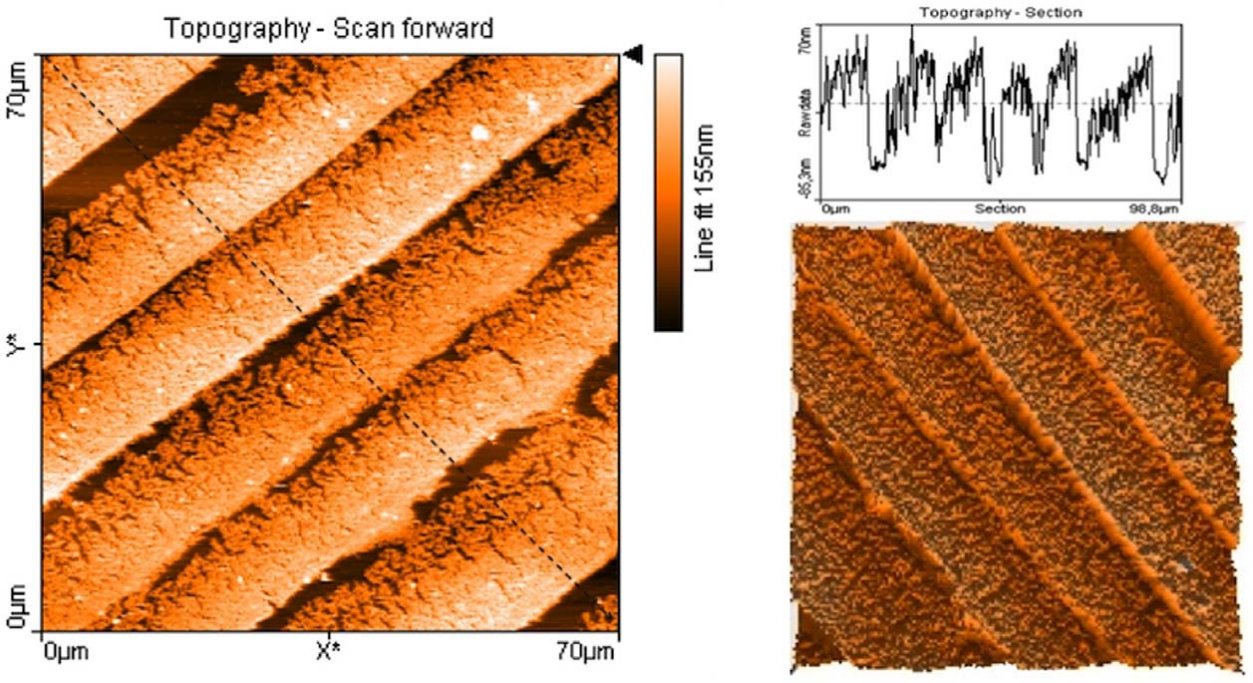
AFM of gold nanobelts imaged with contact mode, showing topographic image (left), topographic section analysis (top right) and 3D mode of topographic image (bottom right). The section analysis on top right was according to the black line in the topographic image.

The SEM images also show that neither the gold nanowire density nor the thicknesses of the gold nanobelts were uniform. One side of the gold nanobelt was found to be thicker than the other side, where also the density of gold nanowires was also higher than the other side. However, these phenomenons aid in the creation of gold nanoparticle patterns that will be discussed below. To measure the thickness of the gold nanobelts, AFM imaging with contact mode was used, as shown in Figure 2. The AFM results gave similar result as SEM, with one side showing higher density. The nanobelts at the high-density sides were thicker, as shown in the AFM cross sension analysis and 3D plotting in Figure 2.

Do discuss the mechanism behind the nanobelt formation we can learn for mseveral similar systems, where the most familiar one is the formation of rings when a drop of colloidal suspension is let to dry, which occur, for example, during the drying of coffee stains [14], [15]. In such drops the evaporation rate is greatest at the edge resulting in a flow of solution toward the edge and thus an increase in the concentration of particles at the edge. In such a droplet experiment, Adachi et al show the formation of pattern of multiple concentric rings of particles [16]. They used suspensions of sub-micron size polystyrene particles forming droplets on glass that was let to dry. They observed an oscillatory motion of he droplet during drying that resembles frictional stick-slip motion, resulting in a pattern of regular concentric rings.

**Figure 3:**
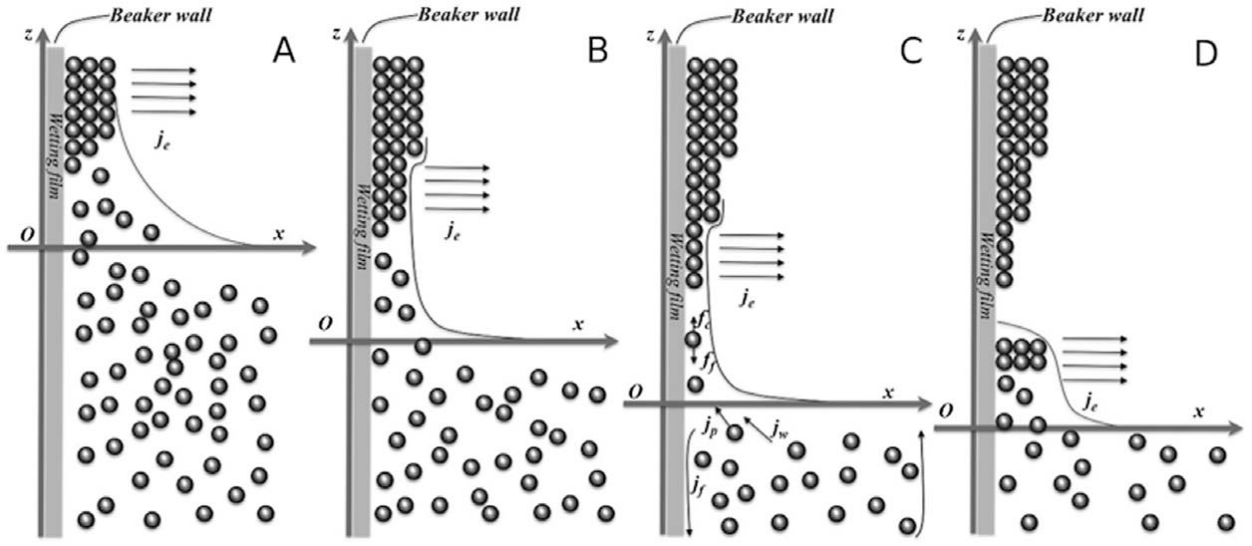
Schematic picture of the growth of gold nanobelts. (A) the beginning of a belt, (B) the density change of nanoparticles on the belt, (C) the formation of a new pinning site, (D) the next belt starts to grow. *j_e_* is the evaporation of water on the wetting film, *j_p_* and *j_w_* are the gold nanoparticles and water influx towards the meniscus driven by evaporation induced capillary force, *f_c_* is the capillary force that drives the nanoparticle to assemble, *f_f_* is the force which drags the nanoparticle down to the bulk solution that is generated by the convection flux.

Using some of the concepts from these drying drop studies we can make a qualitative discussion of the nanobelt formation process and in Figure 3 a simple picture of the process is shown. First, normal EISA deposition occurs, causing deposition of gold nanoparticles. However, since the wetting water film is much thicker that the diameter of the gold nanoparticles, multilayers of particles will be deposited on the beaker wall (Figure 3A) that then will coalesce into gold nanowire networks [12]. When gold is deposited on the glass surface, the surface tension will change from a hydrophobic surface to more hydrophilic one (because the surface of gold nanoparticles here is hydrophilic). This change in surface tension will cause a decrease in thickness of the wetting film and resulting in a reduction of the deposited gold layers (Figure 3 B). This simple picture might provide an explanation of the non-uniformity in thickness of the nanobelts, with one side of the belt thicker than the other one. When the thickness of the deposited gold film is thin enough the bare glass surface will again give hydrophobic surface leading to a new formation of a nanobelt (Figure 3 C) and the process repeats again (Figure 3 D). Although such a picture could provide an explanation, a separate study is needed to provide a quantitative model. We note, for example, that in a model on dry drops due to Adachi et al [16], the number of rings per unit length is inversely proportional to the receding velocity of the drop. To test this in our system, a redesigned set-up is needed to allow a changed in the speed parameter. In our system the speed changes with temperature as a higher temperature lead to higher evaporation rate. We indeed observed a change in the number of nanobelts that is inversely decreasing with temperature. However, higher temperature also increase the convection flow due to a difference in the bath temperature and the surrounding, making it difficult to separate the speed parameter from a temperature effect. Thus, there is one more parameter to add, the convection flux, *j_f_*, (Figure 3 C), since warmer water will flow up from the center of the beaker and colder water will flow down the sides of the beaker wall. One more reason to include the convection flux, is the observation that when the bath was hold at room temperature, a gold film was formed but was not obviously separated into nanobelts (See Figure S4). Which means the convection flux should be a parameter to the separation of the gold belts. A possible effect is that the convection flux generates a force, *f_f_*, which counteracts the force, *f_e_*, generating by the evaporation of water on the wetting film (Figure 3 C).

**Figure 4:**
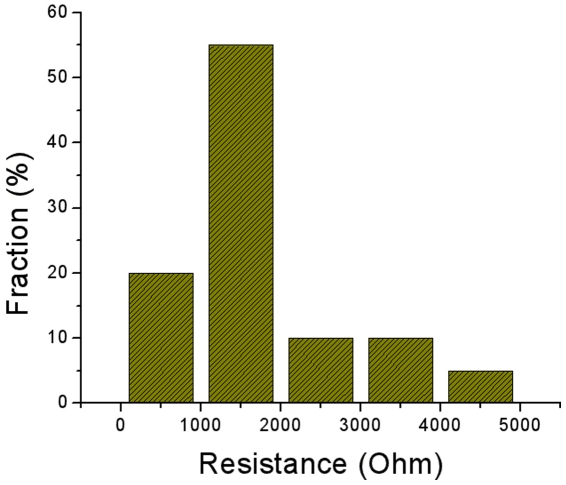
Histogram of the resistance (Ω) distribution of twenty different gold nanobelts, calculated from measured IV data.

These gold nanobelts were found well packed at the onset; however, they can be separated by tapping the beaker wall, allowing them to float on the surface of the solution. The floating nanobelts could then be transferred onto a substrate. The gold nanobelts were barely connected, causing very high resistance between these belts. However, one can measure the resistance of a single gold nanobelt. Figure 4 shows a histogram of the resistance distribution of twenty gold nanobelts calculated from measured IV data, indicating that the most common resistance is around 1000 Ω. However, several nanobelts were found with higher resistance, which was caused by the cracks of the gold nanobelts that occurred during the transfer process (Similar to cracks shown in Figure 1 B).

The structure and the size of the gold nanobelts suggest that they might be useful in the production of electronic devices. On the other hand, they can also be used as a strain sensor, due to the porous structure and the elastic property of the gold nanobelts. We did a simple test model using gold nanobelts film deposited on a rubber wafer to show the viability of the gold nanobelts as strain sensors. We first compressed the rubber wafer from 2.1 cm to 1.9 cm and fixed it in a frame that was controlled by a screw, we then deposited a 1.0 cm×0.1 cm gold nanobelts film on the rubber. After that, we kept three gold nanobelts of the deposited film and cut the others. We used three nanobelts, instead of the ideal case with only one, since it was hard to realize this experimentally with our equipment. Subsequently, the terminals of the gold nanobelts were fixed on the rubber by conductive silver glue for electric measurements.

**Figure 5:**
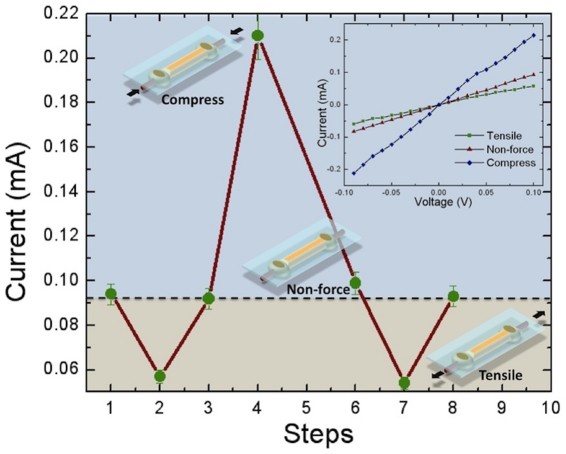
Measured currents on gold nanobelts under different forces. The insert figure shows the measured IV data.

Since the gold nanobelts were deposited on a compressed rubber film, a tensile force can be applied by reducing the compressive force or be compressed by increasing the compressive force. Figure 5 shows the measured currents under compressive and tensile forces as well as no applied force. Results indicated that under tensile force (the rubber was released from 1.9 cm to 2.1 cm), the current decreased from 0.09 mA to 0.06 mA, indicating a 30% reduction. However, the current increased to 0.21 mA under compressive force (the rubber was squeezed from 1.9 cm to 1.72 cm), indicating a 230% increase. The current reduction or increase is due to the density variation of the gold nanobelts. When tensile force was added, the density of gold nanowires in the belts decreased, which in turn decreased the physical contacts between gold nanowires, causing the increase of resistance. Conversely, when compressive force was added, the density increased, which in turn increased the chance for gold nanowire to make contact with each other, causing the decrease of resistance.

Our results demonstrated that porous gold nanobelts could be potentially used as electrical strain sensors [17], although more detailed work is needed. Furthermore, the nanobelts might also be used as optical strain sensors, due to the porous structure. The porous structure of the gold nanobelts makes them transparent, which allows light to pass through. The density variation in the gold nanobelts will change the transparency of the belts; this transparency can be used as a signal for stains.

**Figure 6:**
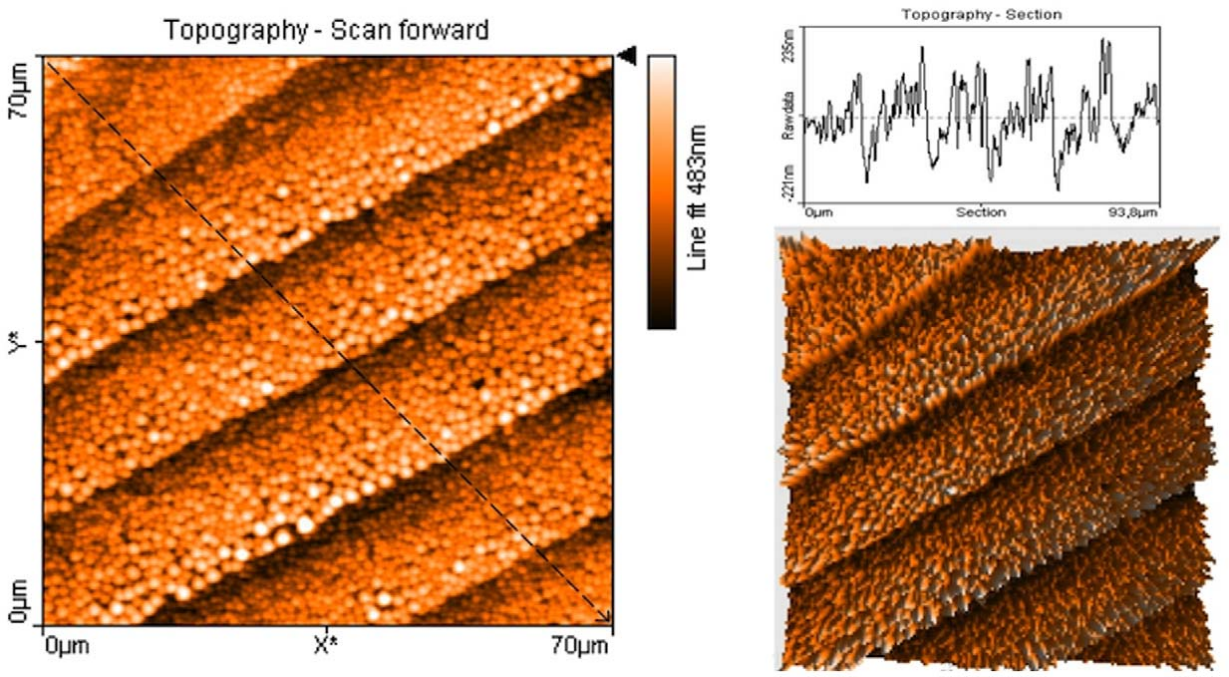
AFM of the gold nanoparticle patterns annealed from gold nanobelts, showing topographic image (left), topographic section analysis (top right) and 3D mode of topographic image (bottom right). The section analysis on top right was according to the black line in the topographic image.

Beside strain sensors, the gold nanobelts can also be used for making gold nanoparticle patterns by annealing the gold nanowires (See schematic drawing of the process in Figure S5). Figure 6 shows an AFM image of the gold nanoparticle pattern made from gold nanobelt annealed at 1100°C. Since the annealing temperature was higher than the melting point of gold, all the gold nanowires in the belt melted and formed gold nanoparticles. These nanoparticles were larger than the original synthesized gold nanoparticles (See SEM images in Figure S6). The size of these dispersed gold nanoparticles after annealing ranged from 70 nm to 700 nm, due to the difference in density between the two sides of gold nanobelts.

**Figure 7:**
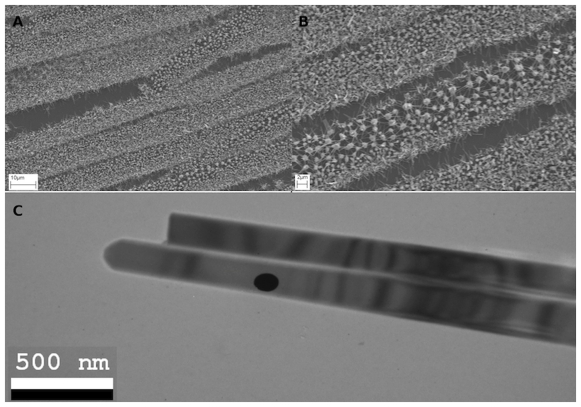
SEM of ZnO nanowires grown on gold nanobelt patterns at low (A) and high (B) magnification; and TEM of the ZnO nanowire (C). The black dot in the TEM images is an artifact for imaging.

Recently, it has been shown that gold nanoparticles annealed from thermal evaporated gold film [18] on silicon wafer can be used to grow nanowires [19], suggesting that our gold nanoparticle patterns, one can grow patterned nanowires. To demonstrate this, we grew ZnO nanowire patterns [20] on the gold nanobelt patterns. Such structures can be used as nano-generators [21], to convert mechanical to electrical energy. Figure 7A and B shows SEM images of grown ZnO nanowires that were patterned by the gold nanoparticles. Different growing behavior was found on the two sides of the gold nanobelts. At one side where the gold nanowires were annealed into small nanoparticles, the grown ZnO nanowires had a higher density than on the other side. Moreover, the nanowires grown at the small nanoparticle side were found to mainly grow vertically, while on the other side where the gold nanoparticles were larger, the nanowires grew horizontally forming networks between the nanoparticles. Figure 7C also shows a TEM image of grown ZnO nanowire with an average diameter of about 150 nm.

The anneal gold nanoparticles patterns (Figure 6) might have potential application in Raman spectroscopy, since gold nanoparticles are enhancing the Raman signals of some molecules [22]. These patterned gold nanoparticles with clean surfaces (all organics have been burned off during the annealing process) might allow patterned Raman signals for investigating molecular structures. Moreover, the advantage of the gold nanoparticles annealed on silicon wafer is that it can be re-used, in contrast to commonly used self-assembled gold nanoparticle film [22]. The annealing at 1100°C formed Au-Si alloy [23] at gold nanoparticle-silicon interface, which makes the gold nanoparticles bind strongly to the wafer so that they can be rinsed with strong acid to remove modified organic molecules. Also, it can be cleaned by burning off the organics.

### Conclusion

In summary, we reported a simple reagent free EISA method to produce well packed porous gold nanobelts with L×W×T of (∼0.15 m)×(∼25 µm)×(∼80 nm). SEM and AFM images showed that gold nanobelts were different in structures on two sides, where the density of gold nanowires on one side was higher than the other side. These uniquely gold nanobelt patterned grown by the EISA method is similar to stick-slip motion induced “coffee rings”, but with a difference in that there was a temperature difference between the solution and the surrounding air. The resistances of 2 mm long gold nanobelts were around 1000 Ω, however, the resistances could be higher due to the cracks in the belts. The porous gold nanobelts allowed a simple demonstration of a strain sensor. Moreover, these gold nanobelts can also be used to produce gold nanoparticle patterns by simply annealing the gold nanobelts above the melting point of gold, and furthermore, the gold nanobelt patterns were demonstrated to be used to grown ZnO nanowire pattern that might be potentially used in the study of piezoelectronics.

## Supporting Information

Figure S1 **(A), (B) and (C) TEM image of as-synthesized gold nanoparticles from different places; (D) shows the electron diffraction pattern of gold nanoparticles.** The black dots in the TEM images are artifacts for imaging.
(TIFF)

Figure S2 **SEM of gold nanobelt film grown at pre-treatment of 40°C.**
(TIFF)

Figure S3 **SEM of gold nanobelt film grown at pre-treatment of 70°C.**
(TIFF)

Figure S4 **SEM of gold nanobelt film grown at room temperature without any pre-treatment.**
(TIFF)

Figure S5 **Schematic drawing of the process to make gold nanoparticle patterns.**
(TIFF)

Figure S6 **SEM of gold nanoparticle patterns annealed from gold nanobelts, showing low (A) and high (B) magnification images.**
(TIFF)
