# Supplementary figures and images for: Simple Fabrication of Gold Nanobelts and Patterns
Source: PLoS One. 2012 Jan 23;7(1):e30469. doi: 10.1371/journal.pone.0030469 (PMC3264570; doi:10.1371/journal.pone.0030469)

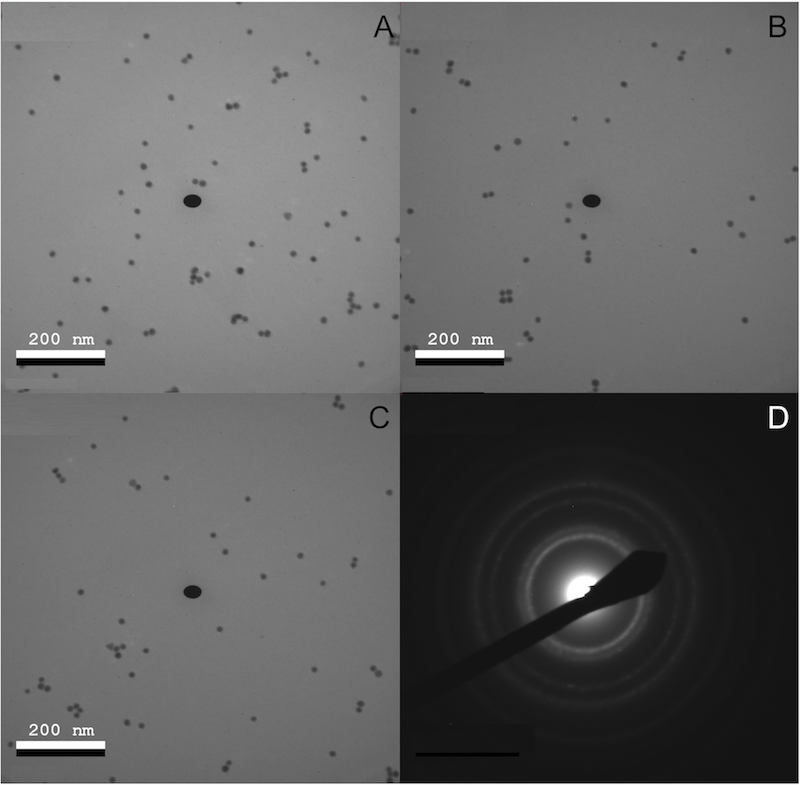

Supplement: Figure S1 — (A), (B) and (C) TEM image of as-synthesized gold nanoparticles from different places; (D) shows the electron diffraction pattern of gold nanoparticles. The black dots in the TEM images are artifacts for imaging. (TIFF) [file pone.0030469.s001.tiff]

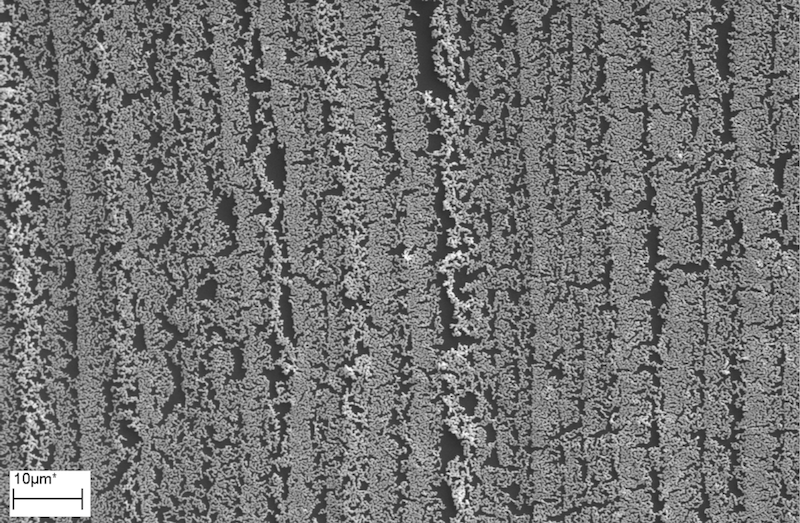

Supplement: Figure S2 — SEM of gold nanobelt film grown at pre-treatment of 40°C. (TIFF) [file pone.0030469.s002.tiff]

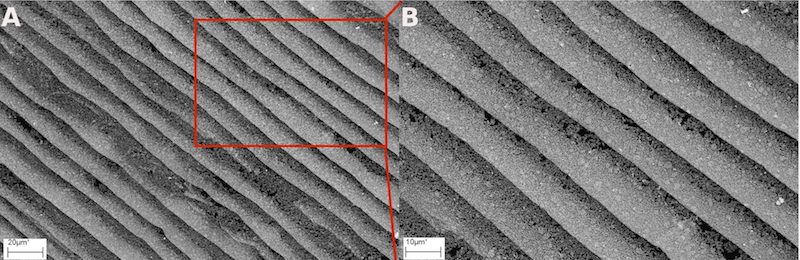

Supplement: Figure S3 — SEM of gold nanobelt film grown at pre-treatment of 70°C. (TIFF) [file pone.0030469.s003.tiff]

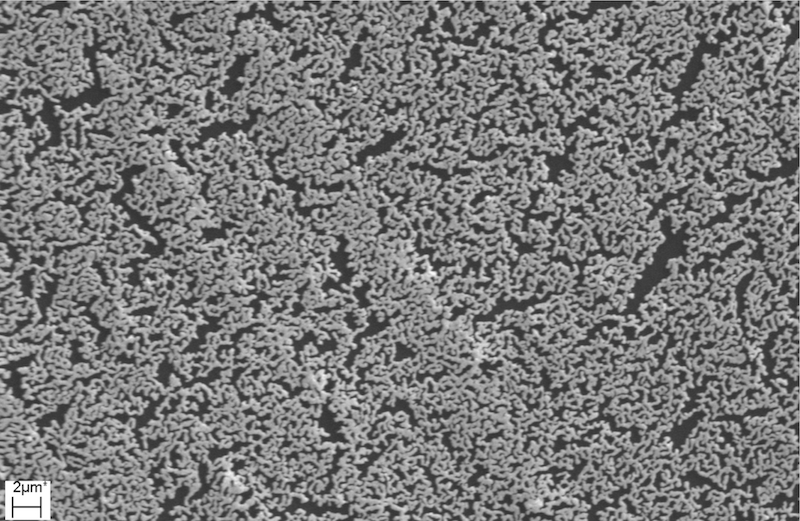

Supplement: Figure S4 — SEM of gold nanobelt film grown at room temperature without any pre-treatment. (TIFF) [file pone.0030469.s004.tiff]

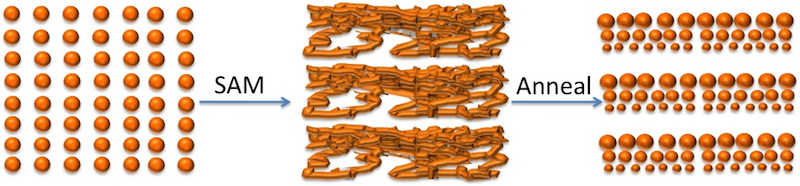

Supplement: Figure S5 — Schematic drawing of the process to make gold nanoparticle patterns. (TIFF) [file pone.0030469.s005.tiff]

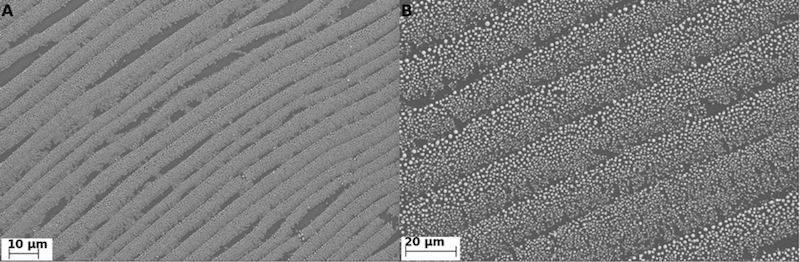

Supplement: Figure S6 — SEM of gold nanoparticle patterns annealed from gold nanobelts, showing low (A) and high (B) magnification images. (TIFF) [file pone.0030469.s006.tiff]
